# Achieving chronic hepatitis B functional cure: Factors and potential mechanisms

**DOI:** 10.1016/j.virusres.2024.199507

**Published:** 2024-12-13

**Authors:** Jiarui Zheng, Zilong Wang, Linxiang Huang, Zixuan Qiu, Yandi Xie, Suzhen Jiang, Bo Feng

**Affiliations:** Beijing Key Laboratory of Hepatitis C and Immunotherapy for Liver Diseases, Peking University People's Hospital, Peking University Hepatology Institute, Beijing, China

**Keywords:** CHB, Functional cure, NAs, PegIFNα, Mechanism

## Abstract

Chronic hepatitis B (CHB) is a significant global health issue affecting approximately 254 million individuals worldwide. Achieving the loss of hepatitis B surface antigen (HBsAg), either with or without seroconversion to hepatitis B surface antibody (HBsAb), is regarded as a functional cure and the optimal goal for addressing CHB, and can be achieved through various approaches, including induction with nucleos(t)ide analogues (NAs), induction with pegylated interferon alpha (PegIFNα), and spontaneous clearance of HBsAg. Spontaneous clearance of HBsAg is rare, while NAs can directly inhibit HBV DNA, they are unable to act on covalently closed circular DNA (cccDNA), hence inhibiting HBsAg production or clearing HBsAg is extremely challenging. On the other hand, functional cure based on PegIFNα shows good long-term durability, but over 10 % of patients still experience relapse, mostly within 48 weeks after functional cure. Factors related to CHB functional cure with antiviral therapy are complex, including host factors, viral factors, environmental factors, etc. The integration of HBV DNA into liver cells, persistence of HBV cccDNA, insufficient B cell responses and compromised T cell function pose significant barriers to HBV clearance. Therefore, this study systematically reviewed the relevant factors and potential mechanisms influencing functional cure CHB, which can provide a basis for personalized treatment, help predict treatment outcomes and assess prognosis, and provide theoretical support for the advancement of novel treatment strategies and medications.

## Highlights

• HBsAg spontaneous clearance of HBsAg is rare, while NAs can directly inhibit HBV DNA, they are unable to act on covalently closed circular DNA (cccDNA), hence inhibiting HBsAg production or clearing HBsAg is extremely challenging.
• On the other hand, functional cure based on Pegifnα shows good long-term durability, but over 10 % of patients still experience relapse, mostly within 48 weeks after functional cure.
• Factors related to CHB functional cure with antiviral therapy are complex, including host factors, viral factors, environmental factors, etc. The integration of HBV DNA into liver cells, persistence of HBV cccDNA, insufficient B cell responses and compromised T cell function pose significant barriers to HBV clearance.
• This study systematically reviewed the relevant factors and potential mechanisms influencing functional cure CHB, which can provide a basis for personalized treatment, help predict treatment outcomes and assess prognosis, and provide theoretical support for the advancement of novel treatment strategies and medications.

## 1 Introduction

Chronic hepatitis B virus (HBV) infection poses a significant public health challenge, presenting a substantial risk to human well-being and serving as a leading contributor to advanced liver diseases, including cirrhosis and hepatocellular carcinoma (HCC), which become evident during the terminal phases of the disease. Based on the most recent data from 2022, it has been observed that the overall occurrence of HBsAg in the broader individuals of China stands at 5.6 %, which indicates that around 80 million people have tested positive for HBsAg (Collaborators, 2023). According to the 2020 Global Cancer Burden Report by the World Health Organization (WHO), there were 906,000 newly diagnosed cases of liver cancer worldwide, leading to 830,000 fatalities, and China contributed to 45.3 % of the new cases and 47.1 % of the deaths (Sung et al., 2021). In contrast to non-HBV-related HCC, HBV-related HCC exhibits shorter survival durations and increased postoperative recurrence rates. WHO has established an objective to eradicate HBV as a public issue by 2030, striving for a 95 % decrease in new instances of CHB and a 65 % reduction in HBV-related fatalities compared to the levels recorded in 2015. As of 2020, China had achieved only a 22 % cumulative diagnosis rate and a 15 % treatment rate for CHB, falling significantly short of the established targets of 90 % and 80 % respectively (Foundation, https://cdafound.org/polaris-countries-dashboard/). Therefore, it is imperative to proactively conduct screenings, provide treatment, and focus on "curing" individuals with chronic HBV infection.

## 2 The definition and clinical significance of CHB functional cure

What does it mean to "cure" a patient with CHB? This requires antiviral therapy to not only achieve the basic endpoint of HBV DNA clearance but also to strive for the satisfactory endpoint of achieving HBeAg seroconversion in individuals diagnosed with HBeAg-positive CHB, and ideally reaching the optimal endpoint of achieving HBsAg seroclearance. The optimal endpoint, known as clinical cure or functional cure, refers to completing a finite course of treatment, achieving HBsAg seroclearance on the basis of undetectable HBeAg and HBV DNA, no matter the presence of anti-HBs, maintaining HBsAg negativity for 24 weeks after treatment cessation, which has become a primary endpoint in evaluating clinical trials of novel therapies for treating CHB (Ghany et al., 2023; Yip et al., 2019).

Domestic and international guidelines have similar goals for the current stage of CHB treatment, all recognizing that only by maximally suppressing HBV replication in the long term, reducing liver cell inflammation, necrosis, and liver fibrosis, can the progression to end-stage liver disease as well as HBV-related mortality be reduced. Different treatment endpoints significantly impact patient prognosis. Research by Yip TC and others found that CHB patients achieving basic, satisfactory, and ideal treatment endpoints had 5-year cumulative HCC incidence rates of 3.6–11.4 %, <2.58 %, and around 1 %, respectively (Yip et al., 2019). Once clinical cure is achieved, the risk of HCC occurrence significantly decreases. A prospective study of CHB patients who achieved HBsAg clearance for up to 12 years showed that after a 4.6-year follow-up period post HBsAg clearance, only 1.1 % of patients progressed to HCC, and 1.3 % of patients experienced liver decompensation. In non-cirrhotic patients, the cumulative incidence rates of HCC were observed to be 0.6 % at 5-year, 0.9 % at 7-year, and 1.5 % at 12-year, respectively, while in cirrhotic patients, these rates were 2.8 %, 3.7 %, and 6.5 % (Yip et al., 2023a). Patients who achieve HBsAg clearance before the age of 50 and do not have cirrhosis have a similar HCC occurrence risk as the general population (Yip et al., 2023b), and the HCC occurrence in individuals who achieve HBsAg clearance through treatment is significantly lower than in those who achieve spontaneous HBsAg clearance (Song et al., 2021).

## 3 Types and epidemiology of CHB functional cure

**Fig. 1:**
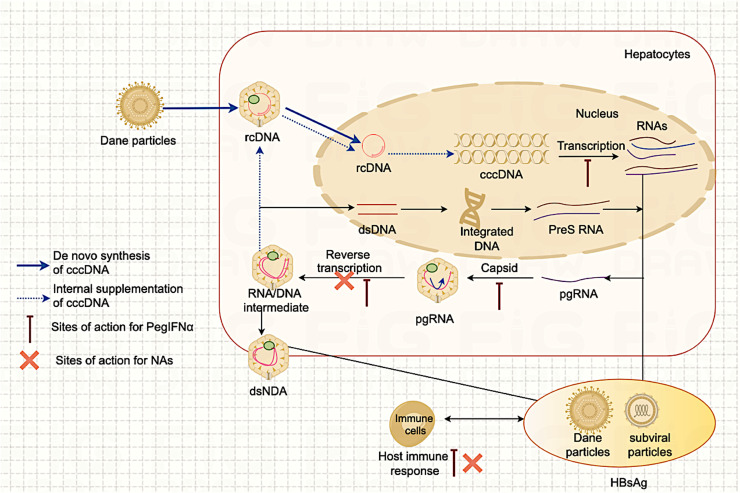
The HBV life cycle and the action sites of NAs and PegIFNα. ccDNA: covalently closed circular DNA; pgRNA: pre-genomic RNA; rcDNA: relaxed circular DNA; dsDNA: double-stranded DNA; NAs: nucleos(t)ide analogues; PegIFNα: pegylated interferon-alpha.

CHB functional cure can be achieved through various methods leading to HBsAg seroclearance, including induction with nucleos(t)ide analogues (NAs), induction with pegylated interferon-alpha (PegIFNα) and HBsAg spontaneous clearance. Fig. 1 depicts the HBV life cycle and the sites of action of NAs and PegIFNα. Spontaneous clearance of HBsAg is rare, with an annual incidence rate of approximately 1 %. NAs directly inhibit HBV DNA but cannot act on covalently closed circular DNA (cccDNA) (Nasser et al., 2023). While NAs alone can achieve virological response, inhibiting HBsAg production or even clearing HBsAg is very challenging (Fung et al., 2022; Jeng et al., 2023; Wang et al., 2023). In a recent large multicenter study involving 4769 CHB patients receiving entecavir or tenofovir antiviral therapy for 26,614 person-years, the 10-year cumulative rate of HBsAg seroclearance was only 2 % (Hsu et al., 2021). Conversely, in CHB patients with sustained virological suppression, some patients may experience seroclearance of HBsAg after discontinuation of NAs. In a multicenter, prospective study analyzing 139 HBeAg-positive, treatment-naive CHB patients without cirrhosis who met the criteria for treatment cessation, the cumulative seroclearance rate of HBsAg after 24 months of cessation was 9.4 % (Xie et al., 2021). In a prospective research involving 1075 HBeAg-negative CHB individuals with an average NA treatment duration of 156 weeks, 5 patients experienced HBsAg seroclearance, at an estimated annual incidence of 0.15 %; among 691 individuals who discontinued treatment according to the APASL guidelines and were followed up for an average of 155 weeks, 42 patients attained HBsAg seroclearance, at an estimated annual incidence of 1.78 % (Jeng et al., 2018). Although PegIFNα has a weak effect on inhibiting HBV DNA, it can effectively reduce HBsAg levels. After treating for 48 weeks, the seroclearance rate of HBsAg reaches 3 %−7 %, and for "advantaged patients," it can even reach 30 %−80 % (Wang et al., 2023).

According to whether HBsAg seroclearance is accompanied by HBsAb seroconversion, it is divided into HBsAg seroclearance and HBsAg seroconversion. He et al. found that in HBV-HIV co-infection individuals receiving antiretroviral therapy (ART) (including TDF and LAM) for an average of 4.7 years, the proportions of patients achieving pure HBsAg seroclearance and HBsAg seroconversion were 49.2 % and 50.8 %, respectively (He et al., 2023).

## 4 Factors and potential mechanisms related to CHB functional cure based on NAs

Factors related to CHB functional cure with antiviral therapy using NAs are complex, including host factors, viral factors, environmental factors, etc. (Table 1). Bruden et al. conducted a study involving 1079 patients with CHB, out of which 134 patients received treatment (67 lamivudine, 42 entecavir, 21 tenofovir, and 4 interferon). After a median 33 years follow-up period, 260 individuals (24 %) attained HBsAg seroclearance (average age at seroclearance was 44.5 years), with an annual clearance rate of 0.82 %. Multivariable analysis revealed that a higher rate of HBsAg seroclearance was linked to older age at the beginning of follow-up, HBeAg negativity, HBV genotypes D and F, and absence of cirrhosis. Due to the limited number of patients receiving antiviral therapy, with only 4 patients having used interferon, the authors did not find a significant impact of antiviral treatment on HBsAg seroclearance rates (Bruden et al., 2024). Huang et al. followed up on 4084 HBeAg-negative CHB individuals and had not received antiviral treatment, of whom 21.7 % had metabolic dysfunction-associated steatotic liver disease (MASLD), and 470 individuals experienced HBsAg seroclearance after a median 5-year follow-up period. Multivariable analysis revealed that factors associated with seroclearance included age ≥50 years, male gender, baseline HBsAg <100 IU/mL, higher ALT levels, presence of MASLD, and cirrhosis. Both HBsAg seroclearance and seroconversion were notably higher in CHB individuals with MASLD in comparison to individuals without MASLD (*p* < 0.001). After adjusting for other factors, MASLD emerged as an independent factor for HBsAg seroconversion (Huang et al., 2024).

**Table 1:** Viral and host factors that associated with NAs/ PegIFNα-induced CHB functional cure.

	Viral and host factors			Role in association with a higher rate of CHB functional cure
NAs	Viral Factors	Viral load		A shorter period to undetectable HBV DNA (Jeng et al., 2018; van Bömmel et al., 2023)
		Genotype and mutants		HBV genotypes B, D and F (Bruden et al., 2024; He et al., 2023; Huang et al., 2024)
		Viral antigen levels	HBsAg	Reduced HBsAg levels at baseline or EOT (van Bömmel et al., 2023; Xie et al., 2021)
			HBeAg	Negative HBeAg at baseline (Bruden et al., 2024)
	Host Factors	ALT		Higher ALT levels (He et al., 2023; Huang et al., 2024; Li et al., 2023)
		Age and gender		Older age and male gender (Bruden et al., 2024; Huang et al., 2024; Xie et al., 2021)
		Liver stage		Presence of cirrhosis and MASLD (Bruden et al., 2024; Huang et al., 2024)
PegIFNα	Viral factors	Viral load		Decreased HBV viral load and antigen burden (Boni et al., 2012; Jiang et al., 2024a; Lebossé et al., 2017; Wu et al., 2024)
		Genotype and mutants		HBV genotypes A (Buster et al., 2009; Jiang et al., 2024b; Marcellin et al., 2016)
		Viral Counteractions	HBsAg	Reduced HBsAg levels at baseline or EOT, swift reduction of HBsAg levels early in treatment (Jiang et al., 2024b; Wu et al., 2020a; Yeo et al., 2019; Yin et al., 2023)
			HBsAb	Higher HBsAb at EOT (Huang et al., 2022; Wu et al., 2020b; Yin et al., 2023)
			HBeAg	Negative HBeAg at baseline (Guo et al., 2023)
			HBcrAg	Lower HBcrAg levels (Hong and Hu, 2021; Hong et al., 2021; Testoni et al., 2019; Yin et al., 2023)
	Host Factors	ALT		Higher ALT levels (Choi et al., 2021a; Perrillo et al., 2022; Zhang et al., 2023)
		Age and gender		Younger age and male gender (Choi et al., 2021b; Jiang et al., 2024b; Jonas et al., 2016; Pan et al., 2022; Wu et al., 2016, 2021)
		SNP		rs7519753, rs7574865, et, al (Balagopal and Thomas, 2024; Chen et al., 2020; Geng et al., 2024)
		Liver stage		Presence of hepatosteatosis (Bacaksız et al., 2021; Li et al., 2022; Mao et al., 2023)

NAs: nucleos(t)ide analogues; PegIFNα: pegylated interferon alpha; EOT: end of treatment; MASLD: metabolic dysfunction-associated steatotic liver disease; SNP: single nucleotide polymorphisms.

He et al. found that in patients co-infected with HBV and HIV receiving antiretroviral therapy (ART) containing TDF and LAM for an average of 4.7 years, the HBsAg seroclearance rate was 8.1 %. Factors associated with this clearance included HBV genotype B, reduced baseline HBsAg levels, and increased ALT levels in the initial six months of ART, while gender, age, ALT levels, baseline CD4+ *T* cell count, and fibrosis degree were not associated with the clearance (He et al., 2023). The occurrence of pre-S mutations and core promoter in HBV genotypes C and D is higher than in genotypes A and B. The pre-core region mutation may diminish the pace of HBsAg seroclearance in therapy, elucidating to some extent the rationale behind the higher seroclearance rate of HBsAg in HBV genotype B. However, the mechanisms behind this require further investigation. Elevated ALT levels may indicate that treatment-naive patients activate the innate immune response via various pathways. The natural immune pathway that secretes pro-inflammatory cytokines could be responsible for flares of ALT. Following antiviral treatment, particularly in patients with well-controlled HBV DNA, there is a boost in the production of pro-inflammatory cytokines and an increase in HBV-specific T cell response capacity (Li et al., 2023). In this scenario, immune response-induced hepatocellular injury may represent immune regulation of HBV. In summary, a potential interpretation is that patients concurrent with HBV and HIV infections undergo an immune reconstitution process following effective ART, subsequently restoring adaptability to HBV medications or innate responses. The recovery of the immune system additionally boosts the ability of HBV-specific T cells to clear infected liver cells, ultimately leading to immune-mediated liver damage or immune reconstitution inflammatory syndrome (He et al., 2023).

Xie et al. found that discontinuation of NAs leading to HBsAg seroclearance in HBeAg-positive CHB individuals was significantly related to age ≥40 years and HBsAg <100 IU/mL at the end of treatment (EOT), and was not related to the duration of antiviral therapy, HBV RNA levels at EOT, or HBcrAg levels (Xie et al., 2021). A multicenter, randomized controlled study on HBeAg-negative CHB patients yielded similar results, showing a significant correlation between HBsAg seroclearance and HBsAg <1000 IU/mL at the end of treatment (van Bömmel et al., 2023). Another study indicated that more substantial HBsAg decline during treatment (>1 log10), lower HBsAg level at EOT (<100 IU/mL), a shorter period to undetectable HBV DNA (<12 weeks), and individuals maintaining a response were determinants for HBsAg seroclearance post-treatment. The rate of HBsAg clearance in clinically relapsed patients without treatment was 7.34 times higher than in patients who received retreatment, suggesting activation of host immunity in patients with transient ALT elevation followed by a decline in viral load and HBsAg levels. Stratified analysis revealed that the seroclearance of HBsAg after treatment cessation in cirrhotic individuals was only associated with "No relapse with no retreatment." While in non-cirrhotic patients, HBsAg seroclearance after treatment cessation was significantly associated with genotype C, the duration for HBV DNA becoming untraceable during treatment <12 weeks, end-of-treatment quantitative HBsAg <100 IU/mL, and no virologic relapse. Patients with sustained response exhibited the highest rate of HBsAg seroclearance, potentially indicating better immune control achieved by these patients before the end of treatment (He et al., 2023). A recent study team dynamically observed distinct T-cell reactions targeting peptides covering the complete HBV proteome in individuals who responded well, experienced relapse, and particularly in those who responded well with HBsAg loss among CHB patients after discontinuation of NAs. The findings demonstrated that CD4^+^ *T* cells specific to HBV played a primary role in HBV-specific T cell reactions and were closely correlated with HBsAg disappearance. The production of IFN-γ by CD4^+^ *T* cells specific to the Envelope protein was heightened in individuals with HBsAg disappearance compared to those positive for HBsAg, and CD4^+^ *T* cells facilitated HBsAg disappearance by boosting humoral immune responses mediated by B cells (Li et al., 2023).

## 5 Factors and potential mechanisms related to CHB functional cure based on PegIFNα

Traditional IFNα has been employed in the management of CHB for over three decades and was substituted by PegIFNα in 2005. Despite its poor tolerability, PegIFNα as the solitary treatment capable of attaining functional cure in individuals with CHB has demonstrated the ability to deliver functional cure to approximately 6–11 % of individuals three years later, especially for those with lower baseline HBsAg levels (<1500 IU/ml), and can increase the functional cure rate from around 5 % to 10 %−30 % (Guan et al., 2024; Ning et al., 2014; Terrault et al., 2018). After achieving clinical cure in CHB patients treated with PegIFNα, in addition to seroconversion of HBsAg, there is a significant reduction in hepatic cccDNA levels and HBV integration levels, with 27 % of patients achieving clearance of cccDNA (Yu et al., 2024). PegIFNα inhibits the entry of pgRNA into the nuclear capsid, reduces the stability of the encapsulated HBV RNA and DNA nuclear capsid, thereby lowering the level of HBV RNA (Mao et al., 2021; Wang et al., 2020); it promotes cccDNA decay and inhibits cccDNA transcription by inducing interferon-stimulated genes (ISGs), facilitating HBV RNA decay and inhibiting its reverse transcription (Imam et al., 2020; Suslov et al., 2018; Yuan et al., 2020). Additionally, as a key cytokine in host antiviral immune responses, PegIFNα exerts a broad impact on various immune cells, encompassing B cells, T cells and NK cells in CHB patients (Meng et al., 2022; Micco et al., 2013; Zhao et al., 2024). However, neither NAs nor PegIFNα can inhibit de novo synthesis of cccDNA or completely block cccDNA replenishment, thus making it impossible to eradicate or completely silence cccDNA (Gan et al., 2023; Yardeni et al., 2023).

### 5.1 Viral factors

Positive aspects of PegIFNα treatment involve a decreased HBV viral load and antigen burden. The viral load can influence both the innate and adaptive immune system pathways, which might impede the response to PegIFNα. Spatial transcriptomics analysis demonstrates that CHB patients who have undergone antiviral therapy exhibit a notable decrease in viral-host chimeric sequences and HBV integration sites compared to treatment-naïve patients. In patients who successfully achieve HBsAg clearance, the presence of chimeric sequences and integration sites is exceptionally rare (Jiang et al., 2024a; Lebossé et al., 2017). Furthermore, the extended exposure of T lymphocytes to a substantial quantity of HBV-associated antigens, particularly HBsAg, is highly probable to be the underlying factor responsible for T cell dysfunction observed in individuals diagnosed with CHB (Fang et al., 2015; Pal et al., 2022, 2019). The reduction in viral load promotes the clearance of antigens and enables T cells to recuperate from antigen stimulation, which could be essential for the restoration of effective T cell responses (Boni et al., 2012; Wu et al., 2024). Moreover, in the primary human hepatocyte (PHH) culture model, increased levels of HBV replication resulted in decreased susceptibility of HBV to PegIFNα (Shen et al., 2018), which aligns with clinical findings that link higher viral load to inadequate response to PegIFNα. This indicates that PegIFNα-based therapy, apart from its immunomodulatory properties, may promote immune system recovery through the direct inhibition of antigen synthesis in liver cells infected with the virus.

Recently, many studies have shown that HBV RNA, as an alternative indicator reflecting cccDNA transcriptional activity, can predict the clearance of HBsAg and serological conversion in patients undergoing PegIFNα therapy. A study including 176 HBeAg-positive patients and 103 HBeAg-negative patients demonstrated that patients achieving an HBV RNA response (defined as a decline in HBV RNA > 2 log10 IU/ml or a decline >1 log10 IU/ml and below the detection limit after 24 weeks of PEG-IFN-α treatment) had a significantly higher rate of subsequent HBsAg seroclearance compared to those who did not achieve an HBV RNA response (10.4% vs. 0.8 %). Furthermore, among patients with an HBV RNA response accompanied by a decrease in HBsAg >1 log10 IU/ml, the rate of HBsAg seroclearance was even higher (28.6 %) (Brakenhoff et al., 2021). Another study involving 133 HBeAg-negative CHB patients showed that patients with lower serum HBV RNA levels at 12 weeks of PegIFNα treatment were more likely to achieve HBsAg seroclearance or a decrease in HBsAg (Farag et al., 2021).

HBsAg level was also independently associated with HBsAg seroclearance as evidenced by numerous studies. The decreased HBsAg levels at the beginning of treatment or EOT, along with the swift reduction of HBsAg levels early in treatment, are strongly linked to an increased likelihood of HBsAg seroclearance (Jiang et al., 2024b; Wu et al., 2020a; Yeo et al., 2019). HBsAb has the capacity to offer sustained protection in the ordinary population by facilitating the removal of circulating HBsAg through antibodies (Hehle et al., 2020). After receiving bone marrow containing HBsAb, a considerable proportion of bone marrow recipients achieved seroconversion of HBsAg or even serological conversion (Lee et al., 2020). In a limited population, PegIFNα-based therapy could lead to the development of HBsAb seroconversion (Cao et al., 2017). A pilot study including 80 patients found that higher HBsAb at EOT were related to sustained response, with AUROC of 0.744, and a combination of HBsAb >2 log_10_IU/L and HBcrAg <4 log_10_U/ml at EOT was associated with a 100 % positive predictive value for sustained response, with AUROC of 0.822 (Huang et al., 2022). HBsAb are theoretically produced by HBsAb-specific B cells that include antibody-secreting cells and memory B cells. A recent study found that patients with baseline HBsAg ≤ 1500 and HBsAb-specific B cells had a higher HBsAg clearance at EOT (Yin et al., 2023).

Nowadays, many studies have proven Hepatitis B core-related antigen (HBcrAg) levels could independently predict HBsAg loss (Huang et al., 2022; Lim et al., 2021). HBcrAg is composed of three interconnected proteins with a common 149-amino acid sequence, comprising the hepatitis B core antigen, HBeAg, and the p22cr protein encoded by the precore/core gene of the Hepatitis B Virus (Inoue et al., 2023; Rokuhara et al., 2003). In individuals with CHB who have not received treatment, the levels of HBcrAg showed a positive association with serum HBV DNA, HBsAg, HBeAg, and cccDNA levels. HBcrAg might provide a more precise indication of cccDNA transcription activity compared to HBsAg (Erken et al., 2021; Testoni et al., 2019), could predict treatment response and relapse following treatment cessation (Hsu et al., 2019; Tsai et al., 2024). Levels of HBcrAg were notably elevated, yet displayed a more substantial decline in individuals positive for HBeAg compared to those negative for HBeAg, possibly due in part to the inclusion of HBeAg as a component of HBcrAg (Hong and Hu, 2021; Hong et al., 2021; Testoni et al., 2019), while other research has also indicated that serum HBcrAg concentrations were markedly elevated in individuals positive for HBeAg compared to those negative for HBeAg, in the absence of antiviral therapy (Chen et al., 2017; Maasoumy et al., 2015). HBcrAg has also demonstrated predictive value for virologic relapse in individuals without HBeAg (Huang et al., 2022; Jung et al., 2016). Nevertheless, the exact underlying mechanism remains unclear and requires further exploration.

### 5.2 Host factors

Host factors, including age, gender, host genetic single nucleotide polymorphisms (SNPs) and host immune status, can influence the rate of functional cure based on PegIFNα therapy. Compared to adults, antiviral treatment in children with CHB can achieve a more rapid and effective serological conversion of HBeAg and HBsAg. As the first choice for the antiviral treatment of CHB in children, multiple studies have shown that clinical cure rates exceeding 50 % can be achieved in CHB children who undergo treatment with PegIFNα. Furthermore, younger age is associated with higher cure rates, indicating significant long-term benefits (Jonas et al., 2016; Pan et al., 2022; Wu et al., 2016). In addition to age, studies have shown that male patients are more likely to achieve functional cure. Choi HSJ, et al. found that male as a baseline factor was associated with a higher frequency of HBsAg clearance when contrasted with females (Choi et al., 2021b). Wu Y, et al. also showed that among PegIFNα- treated CHB patients who achieved function cure during the 2-year follow up, males accounted for a higher proportion (Wu et al., 2021).

SNP also play a key role on PegIFNα-based functional cure. In recent research utilizing a genome-wide association study (GWAS) to analyze DNA sequences, researchers identified rs7519753 which locus at chromosome 1q41 (Guan et al., 2024). The presence of at least one C allele at rs7519753 was observed in 58.3 % of individuals in the HBsAg clearance group, contrasting with only 6.4 % in the non-responsive group to PegIFNα. This discovery was further validated by genotyping rs7519753 in two separate cohorts treated with PegIFNα: comprising 90 HBsAg clearance cases and 134 non-clearance cases. In both validation sets, the C allele was notably more prevalent in individuals who achieved HBsAg clearance, and individuals with a C allele were 2.3 times more likely to achieve HBsAg clearance with PegIFNα treatment. Furthermore, the rs7519753 SNP exhibited strong linkage disequilibrium with several other SNPs that were predicted to regulate TP53BP2 (Balagopal and Thomas, 2024). An additional retrospective review involving 1823 individuals with chronic HBV infection and HBeAg positivity (954 patients receiving PegIFNα and 869 patients receiving NAs) from four phase-4 multicenter randomized controlled trials revealed a notable correlation between the STAT4 rs7574865 genotype and complete remission (CR, *p* = 0.004) as well as HBsAg clearance (*p* = 0.037) in PegIFNα-treated patients. Conversely, no significant association was observed between the rs7574865 genotype and CR (*p* = 0.811) or HBsAg clearance (*P* = 0.439) in patients treated with NAs (Chen et al., 2020). A recent prospective case–control study including 131 PegIFNα-treated CHB patients showed that PAK4 rs9676717, CYP27B1 rs4646536 and IL28B rs12979860 were independently associated with HBsAg clearance. Furthermore, the model that included rs4646536, rs3077, rs12979860, rs9676717, rs2850015, baseline HBsAg, HBeAg status, and HBV DNA had the best prediction performance for HBsAg clearance prediction, with AUC = 0.877 (Geng et al., 2024).

The integration of HBV DNA into liver cells, persistence of HBV cccDNA, insufficient B cell responses and compromised T cell function pose significant barriers to HBV clearance. These factors establish that CHB cannot be eradicated entirely like chronic hepatitis C through direct antiviral medications. CD8+ *T* lymphocytes specific to HBV are pivotal in regulating the replication of HBV by eliminating infected liver cells via cytotoxic T cell-induced cell death and through noncytolytic mechanisms mediated by TNFα (Phillips et al., 2010; Takahama et al., 2023). Additionally, the responses of Tfh and B cells could boost the production of HBsAb and facilitate the elimination of HBsAg (Poonia et al., 2018; Wang et al., 2018). Hence, to effectively treat CHB, it is crucial to enhance the host's immune function alongside suppressing viral replication and decreasing, or potentially eradicating, viral antigens (Chang and Liaw, 2022).

### 5.3 Factors and potential mechanisms related to CHB functional cure based on different antivral treatment strategy

Regimens based on PegIFNα present numerous disadvantages: extended therapy durations, inconvenient dosing schedules, severe side effects, and low functional cure rate. Enhancing PegIFNa itself (prolonged half-life, enhanced hepatocyte targeting, reduced and milder adverse effects) and refining PegIFNa-based treatment protocols (increased functional cure rates) stand out as key avenues for future advancements (Chen et al., 2021). In the past 20 years, different combined approaches to antiviral therapy using NAs and PegIFNα have been explored to enhance outcomes such as achieving undetectable HBV DNA, normalizing ALT levels, achieving loss of HBeAg and loss of HBsAg. Key combination strategies involve initiating therapy with both NAs and PegIFNα, transitioning from NAs to PegIFNα or vice versa, and combining PegIFNα with NAs or vice versa (Fonseca et al., 2020; Lee et al., 2021; Lim et al., 2022; Liu et al., 2020). The combination of PegIFNα with NAs treatment has a notable impact on the activity and characteristics of NK cells, contributing significantly to the clearance of HBsAg. In a study by Stelma et al., CHB patients undergoing a 48-week regimen of PegIFNα combined with adefovir exhibited a substantial rise in both the absolute count and percentage of CD56^bright^ NK cells, alongside a reduction in CD56^dim^ NK cells among those receiving the combined therapy (Stelma et al., 2015). Individuals classified as inactive carriers of HBsAg (IHC), traditionally not deemed in need of treatment, can experience an increased rate of HBsAg seroconversion following PegIFNα therapy.

Prolonging the course of PegIFNα treatment may potentially increase the clinical cure rate. However, it does not mean that the treatment should be extended indefinitely in one continuous session without limitations. A phased treatment approach with an overall extended duration may be a better strategy. Research has shown that long-term PegIFNα therapy can lead to CD8+ *T* cell exhaustion, which hinders the achievement of sustained immune response. Therefore, pausing PegIFNα treatment is beneficial for the restoration of host immune function. Simultaneously, maintaining NAs treatment can assist in better reconstruction of specific immunity while ensuring sustained control of HBV DNA, which creates an opportunity for the subsequent use of PegIFNa (Micco et al., 2013; Thimme and Dandri, 2013).

In addition to NAs, PegIFNα combined with other novel drugs also showed important contribution to the clearance of HBsAg. Bulevirtide, a man-made myristoylated peptide, possesses the same 47-amino acid sequence as l-HBsAg essential for NTCP binding, functioning through competitive blocking of the NTCP receptor (Kang and Syed, 2020). In a study involving individuals with HBV and hepatitis D virus co-infection, 26.7 % of participants (4 out of 15 individuals) attained HBsAg elimination following treatment with 2 mg of bulevirtide in combination with PegIFNα by the 72nd week. Interestingly, none of the subjects who received a higher dosage of bulevirtide (5 mg) alongside PegIFNα exhibited HBsAg clearance (Wedemeyer et al., 2019). Considering that only four co-infected individuals experienced HBsAg clearance, it is essential to conduct more extensive trials involving mono-HBV-infected patients to validate these findings.

## 6 Factors and potential mechanisms related to the recurrence of CHB after functional cure

Patients with CHB who achieve functional cure experience a more durable treatment response, with stable disease condition and rare relapse after discontinuation of medication (Alawad et al., 2020; Wu et al., 2020b). Virological relapse is characterized by the resurgence of detectable HBV DNA during post-treatment observation, often accompanied by concurrent seroconversion of HBsAg. Some patients exhibit transient low-level HBV DNA seroconversion, while others demonstrate persistent and gradually increasing HBV DNA seroconversion. Jiang et al. found that the 5-year HBsAg reactivation rate in CHB patients treated with follow-up PegIFNα reached 27.6 %, which is significantly higher than the 3.3 % in the NAs induction group and 8.1 % in the spontaneous HBsAg clearance group. Factors influencing relapse include anti-HBs, anti-HBc, and HBeAg status at EOT (Jiang et al., 2023). Another study on the factors influencing the persistence of HBsAg clearance and HBsAg reactivation in patients treated with PegIFNα also showed the following results: during the 12 months follow-up period, 15.2 % of individuals experienced HBsAg reactivation, which was found to be associated with anti-HBc levels, anti-HBs levels and HBeAg status at EOT. Among HBeAg-negative individuals with anti-HBs levels ≥ 20 IU/mL at EOT, the rate of HBsAg reactivation was only 2.8 % (Guo et al., 2023). Wu et al. conducted a study including 73 patients who achieved HBsAg clearance through PegIFNα treatment indicated that the HBcAb levels at both baseline and EOT were notably higher in the non-relapse group in comparison with the relapse group (*p* = 0.023). Multivariate analysis indicated that solely the HBcAb level at EOT was independently associated with relapse (Wu et al., 2021). A meta-analysis also demonstrated that the rate of relapse in individuals who achieved HBsAg seroconversion accompanied by the appearance of anti-HBs was significantly lower compared to those who remained consistently negative for anti-HBs (Song et al., 2021).

## 7 Clinical significances of the exploration of factors and potential mechanisms of CHB functional cure

cccDNA may be silenced but HBsAg may continue to persist in serum due to production from integrated HBV DNA, which is a limitation of the definition of functional cure (Meier et al., 2021). However, by exploring the relevant factors and potential mechanisms for CHB functional cure, we can increase the influence of favorable factors and reduce the impact of unfavorable factors, which is helpful in guiding the development of clinical treatment strategies. For example, early treatment can increase the chances of functional cure as age is an important factor. Additionally, factors such as viral load levels, immune status and genetic factors may also influence the outcome of CHB functional cure (Tout et al., 2020). By conducting in-depth research on these factors and mechanisms, it is possible to provide clinicians with evidence for selecting the best treatment plan and offer personalized treatment strategies for patients. Furthermore, exploring the mechanisms of CHB functional cure contributes to the development of new treatment methods and drugs. Understanding the replication and transmission mechanisms of the HBV in the body, as well as the reaction of the immune system to the virus, may offer a theoretical foundation for the advancement of new antiviral drugs and immune modulators. These new treatment methods and drugs may be more effective in suppressing viral replication, enhancing immune response, and ultimately promoting the achievement of CHB functional cure.

## 8 Summary and future prospects

In summary, the study of factors and potential mechanisms related to functional cure in CHB is of great clinical significance. These studies can provide a basis for personalized treatment, help predict treatment outcomes and assess prognosis, and provide theoretical support for the development of new treatment strategies and medications. Achieving functional cure in CHB also contribute a positive influence on patients’ life quality and prognosis. Future research should delve deeper into these factors and more large prospective studies focusing on the development of new antiviral drugs, immunotherapy, early diagnosis and intervention as well as personalized treatment should be conducted to develop targeted interventions aimed at achieving functional cure in CHB patients.

## CRediT authorship contribution statement

**Jiarui Zheng:** Resources, Project administration, Methodology. **Zilong Wang:** Writing – original draft. **Linxiang Huang:** Writing – review & editing. **Zixuan Qiu:** Methodology. **Yandi Xie:** Resources. **Suzhen Jiang:** Validation. **Bo Feng:** Supervision, Funding acquisition.

## Declaration of competing interest

The authors declare that they have no known competing financial interests or personal relationships that could have appeared to influence the work reported in this paper.

## Data Availability

No data was used for the research described in the article.
